# Foreword to the focus issue: materials and technologies for memristors and neuromorphic devices

**DOI:** 10.1080/14686996.2023.2263265

**Published:** 2023-10-16

**Authors:** Ye Zhou, Su-Ting Han

**Affiliations:** a Institute for Advanced Study, Shenzhen University, Shenzhen, China; b College of Electronics and Information Engineering, Shenzhen University, Shenzhen, China

As the demand for more advanced artificial intelligence becomes increasingly urgent, scientists gradually realize that von Neumann computers still lag behind the human brain in terms of adaptability, fault tolerance and generalization capabilities. The continuous exploration of the working mechanism of the human brain has given rise to research on efficient neuromorphic computing. The realization of this goal can be achieved by neuromorphic electronics, which aims to realize computing functions by simulating neural behavior with neuromorphic devices. With the iteration of semiconductor technology, neuromorphic devices such as memristors have brought researchers the dawn of efficient neuromorphic computing. Owing to the extensive selectivity of materials and technologies, developing memristors and other neuromorphic devices has been a very promising option to meet the request for future electronics. There still exist challenges in designing high-performance memristors and neuromorphic devices and it is hoped that more novel working mechanisms and devices with functional materials can be found.

This Focus Issue on ‘Materials and Technologies for Memristors and Neuromorphic Devices’ organizes very high-quality review articles on the latest progresses in neuromorphic computing. Memristors and synaptic transistors have been widely studied in neuromorphic devices. Recent research interests include finding suitable materials and structures for mimicking biological nervous systems and developing low-power brain-like algorithms. With the combination of hardware, the functional simulation of the human brain neural network can be realized at the lowest power consumption and cost.

Li et al. [1] from Huazhong University of Science and Technology review the memristor-based neurons and related applications in neuromorphic sensing and computing systems. They also discuss the challenges of the memristor-based neurons toward high-performance neuromorphic systems. Zhang et al. [2] from Nankai University describe the recent breakthrough of graphdiyne in device applications. Graphdiyne-based data storage and neuromorphic devices, together with their applications in artificial visual systems and neuromorphic computing, have been reviewed, which can broaden the application fields of graphdiyne. Zhang et al. [3] from Central South University review the visual bionic applications of memristors and synaptic transistors based on photosensitive materials. They also summarize the problems and challenges of optoelectronic neuromorphic devices, and propose the future growth of visual bionics. Xiao et al. [4] from Hubei University present a review of resistive switching materials including perovskites, binary oxides, two-dimensional materials and organics. They discuss the resistance modulation of the memristor and how to enhance the device performance with proper approaches. Multi-sensory fusion and system-level optimization are also discussed in their paper. Chen et al. [5] from Henan University provide a comprehensive review of brain functions mimicked by neuromorphic devices. Some useful guidelines for exploring synapses, neurons and intelligent behaviors are introduced. Fu et al. [6] from Nanjing University of Posts & Telecommunications review the recent developments of pseudo-transistor-based neuromorphic electronics. The authors comprehensively discuss the materials, device structures and working mechanisms of three typical pseudo-transistors, including memflash, tunneling random access memory and memtransistor. Sun et al. [7] from Ningbo Institute of Materials Technology and Engineering introduce the materials, operating principle and performances of electrolyte-gated transistors. The recent developments of electrolyte-gated transistors for artificial synapses and neurons are also discussed. Wang et al. [8] from Ningbo University review the functions, operation mechanisms and applications of transistor-based neuromorphic devices. The authors provide the latest progresses, the opportunities and the challenges of artificial perception systems. Jiang et al. [9] from Fudan University present a comprehensive review of phase-change memory materials including the thermal, structural and electrical dynamics. The authors also suggest a prognosis on the unsolved challenges in phase-change memory.

As guest editors, we would like to thank all authors and reviewers who have contributed to this Focus Issue. We believe that the nine review papers in this STAM Focus Issue can provide the latest advancements in memristors and neuromorphic devices. We hope this Focus Issue can inspire numerous outstanding developments and new ideas in the neuromorphic computing research area and offer some strategies for the future development of memristive materials in neuromorphic electronics.
